# Cyclodextrin-Containing Drug Delivery Systems and Their Applications in Neurodegenerative Disorders

**DOI:** 10.3390/ijms251910834

**Published:** 2024-10-09

**Authors:** Yuan Xing, Bohan Meng, Qi Chen

**Affiliations:** 1 MOE Key Laboratory for Analytical Science of Food Safety and Biology, College of Chemistry, Fuzhou University, Fuzhou 350108, China; 15725380186@163.com (Y.X.); 15206670218@163.com (B.M.); 2 Interdisciplinary Institute for Medical Engineering, Fuzhou University, Fuzhou 350108, China

**Keywords:** cyclodextrin, neurodegenerative disorders, drug delivery systems

## Abstract

Cyclodextrins (CDs) are ubiquitous excipients, constituted of cyclic glucopyranose units, and possess a unique dual nature, that of a hydrophobic interior and a hydrophilic exterior. This enables their interaction with lipid-affinitive compounds and hydrophilic compounds, thereby augmenting their application in pharmaceutical formulations as agents for improving solubility, as well as fundamental elements of advanced drug delivery systems. Additionally, CDs, upon suitable modification, can strategically participate in the interaction with cellular components and physical barriers, such as the blood–brain barrier, where their intricate and multifunctional engagement leads to various biological impacts. This review consolidates the crucial features of CDs and their derivatives, and summarizes the applications of them as drug delivery systems in neurodegenerative disorders, emphasizing their notable potentials.

## 1. Introduction

Neurodegenerative disorders impact a population exceeding one billion individuals globally [1]. The rise in life expectancy coincides with a surge in the prevalence of chronic, age-associated neurodegenerative disorders, notably Parkinson’s disease (PD), Alzheimer’s disease (AD), and so on [2]. The progression rates of neurodegenerative disorders are hastened by oxidative stress and inflammatory responses [3,4], leading to mitochondrial dysfunction [5,6], cellular injury, and disruptions in DNA repair systems [7,8]. The existence of the blood–brain barrier (BBB) poses additional hurdles, necessitating the urgent development of novel therapeutic approaches for neurodegenerative disorders [9]. Moreover, a majority of these disorders arise from the accumulation of lipids, such as cholesterol [10].

Nanoassemblies have garnered significant interest in biomedical fields due to their multifaceted utilization, particularly in the realm of targeted and regulated drug release [11]. Cyclodextrin (CD) stands as a prominent example in this arena. For many decades, it has been extensively and effectively utilized, primarily to augment the solubility, stability, and bioavailability of numerous therapeutic agents [12,13]. Similarly, interlocked molecular structures based on CDs, including rotaxanes, polyrotaxanes, catenanes, and polycatenanes, have been fabricated and have garnered significant interest [14,15]. Noteworthy, a number of CDs display not only neuroprotective characteristics but also regulate cholesterol metabolism within cortical neuron cultures [16]. Specifically, they demonstrate the capacity to extract cholesterol from cellular membranes, thereby alleviating its accumulation [17,18]. As an illustrative instance, methyl-β-cyclodextrin (M-β-CD) and hydroxypropyl-β-cyclodextrin (HP-β-CD) have been shown to effectively mitigate lysosomal cholesterol build-up in primary neuronal and glial cells originating from Niemann–Pick type C1 (NPC1) mutant mice [19]. Thus, studying and understanding the properties of CDs, and exploring CD-based drug delivery systems for neurodegenerative disorders holds significant research value.

## 2. Cyclodextrin (CD)

CD can be formed through starch fermentation processes carried out by specific bacterial strains, while industrially, they are mass-produced via the action of the enzyme CD-glycosyltransferase, which catalyzes starch breakdown and subsequent ring formation [20,21]. It possesses a hollow truncated cone structure characterized by a hydrophobic inner cavity and a hydrophilic outer surface. Hydrophobic drugs can be encapsulated within the CD cavity via noncovalent interactions, resulting in the formation of inclusion complexes without the need for complex chemical reactions. Hence, water solubility and the stability of drugs can be greatly improved. Additionally, CD-based polymers or assemblies are capable of condensing DNA and RNA, which can then be utilized as genetic therapeutic agents [22].

The US Food and Drug Administration (FDA) has approved HP-β-CD as an adjuvant of itraconazole injection for the treatment of systemic fungal infections, and docetaxel injection (driven by sulfobutyl-β-CD) for the treatment of breast cancer, non-small-cell lung cancer, gastric adenocarcinoma, hormone-refractory prostate cancer, and head and neck squamous cell cancer.

As previously introduced, since CD extracts cellular cholesterol and mitigates neuronal accumulation [23], the FDA has approved HP-β-CD for the treatment of Niemann–Pick Disease Type C (NPC) in twin sisters, which is referred to as ‘pediatric Alzheimer’s’ [24]. Wilson et al. evaluated the safety and effectiveness of intrathecal injection of HP-β-CD into the lumbar spine. Patients with NPC1 who received an intrathecal injection of HP-β-CD slowed down the progress of the disease, and the safety was acceptable. These results support the initiation of a multinational, randomized, and controlled trial of intrathecal injection of HP-β-CD [25]. Recently, Sharma et al. evaluated the safety, tolerability, and efficacy across multiple clinical endpoints following intravenous administration of HP-β-CD in NPC1 patients. In pediatric and adult patients, HP-β-CD alleviated clinical symptoms and was generally well-tolerated [26].

HP-β-CD, which consists of seven glucose molecules [27], engages with amyloid-beta (Aβ), markedly diminishing its toxic impact in both cell and animal studies [28]. Below are examples of studies on CDs and their derivatives in neurodegenerative diseases. Yao et al. explored the effects of HP-β-CD in cellular and mouse models of AD [29]. They observed that HP-β-CD reduced cholesterol accumulation in N2a cells overexpressing the Swedish mutant form of amyloid precursor protein (SwN2a) and significantly lowered Aβ42 levels. In Tg19959 mice, a transgenic model of AD, four months of subcutaneous HP-β-CD administration improved spatial learning and memory deficits, decreased Aβ plaque deposition, and reduced tau-immunoreactive dystrophic neurites. HP-β-CD reduced Aβ42 levels by inhibiting β-secretase-mediated cleavage of amyloid precursor protein and upregulating genes involved in cholesterol transport and Aβ clearance. These findings suggest a novel therapeutic strategy for AD by both reducing Aβ production and enhancing its clearance. Kilpatrick et al. demonstrated that chemical activation of transcription factor EB using HP-β-CD facilitates the autophagic clearance of aggregated α-synuclein (α-syn) [30]. These findings support the role of transcription factor EB as a therapeutic target for the treatment of PD and potentially other neurodegenerative diseases characterized by protein aggregation.

In addition to the direct use of CDs, they can also be designed as functionalized systems for the treatment of neurodegenerative diseases. Since the discovery in 1992 of a novel structure assembled via the host–guest interaction between α-CD and polyethylene glycol (PEG), there have been a plethora of reports on its applications in functional systems [31]. In the field of biomedical engineering, α-CDs have been pre-assembled into “molecular abacuses” [32], subsequently functionalized to modify polyethylenimine (PEI) and drugs, enabling gene or drug delivery [33,34]. Similarly, Puglisi et al. developed a novel β-CD-appended polymeric system, based on a pH-cleavable benzoic-imine linkage [35]. The conjugate self-assembled into polymersome microparticles, exhibiting stability at physiological pH 7.4 and rapid hydrolysis at pH 5.5. Studies in human umbilical vein endothelial cells confirmed non-toxicity and rapid cellular uptake within 30 min. This β-CD-functionalized polymer represents a promising tool for developing therapies targeting lysosomal cholesterol reduction.

**Figure 1:**
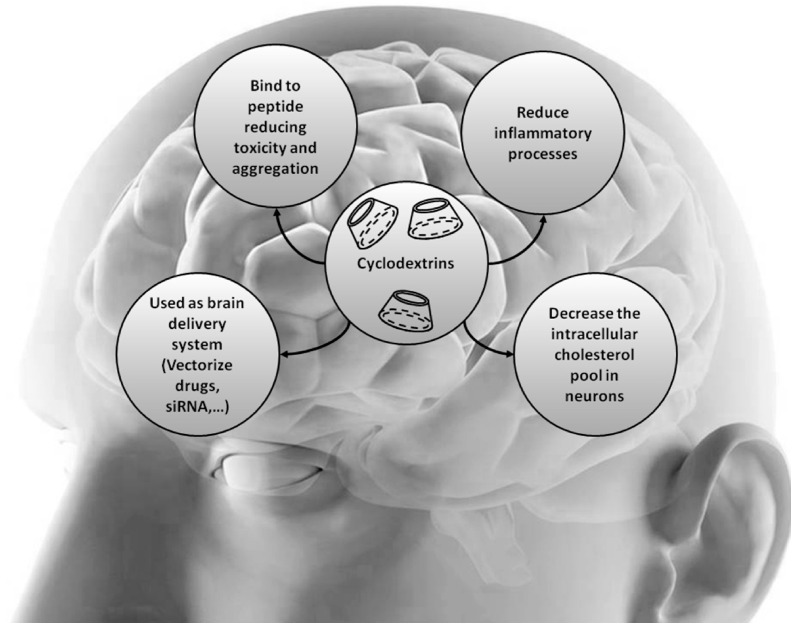
CDs used as brain delivery systems exert influences at multiple stages, including binding to peptides reducing toxicity and aggregation, reducing inflammatory processes, and decreasing the intracellular cholesterol pool in neurons, demonstrating therapeutic benefits in neurodegenerative conditions. Reproduced with permission from [36].

Thus, both the direct and indirect impacts of the CDs have demonstrated efficacy, and numerous research methodologies continuously assess their potential for therapeutic applications in neurodegenerative diseases (Figure 1) [36].

## 3. Neurodegenerative Disorders

The escalating incidence of neurodegenerative conditions can partly be attributed to advancements in longevity. Regrettably, despite concerted efforts, a definitive cure for these ailments remains elusive at present [37].

### 3.1. Challenges in the Treatment of Neurodegenerative Diseases

The term neurodegenerative disorder covers a wide range of diseases [38]. Numerous neurodegenerative disorders remain less recognized and studied extensively [39]. There are several common features (pathological characteristics and biological pathways) that overlap among neurodegenerative disorders [40], with the most significant shared feature being the loss of neurons located in the cortical and hippocampal areas in AD, the striatal areas in PD, and the cortical and striatal areas (specifically medium spiny neurons) in Huntington’s disease (HD) [41]. Additionally, multiple sclerosis (MS) targets the myelin sheath of central nervous system (CNS) neurons, leading to axon injury and neuronal death [42], while amyotrophic lateral sclerosis (ALS) is characterized by the progressive degeneration of motor neurons in the brain and spinal cord [43].

The high degree of overlap in clinical phenotypes makes accurate diagnosis or differentiation between disorders extremely difficult [44]. For example, AD and frontotemporal dementia may present with similar cognitive and behavioral symptoms in their early stages [45].

Effective treatments are presently absent for most neurodegenerative disorders [46]. For certain conditions, like Parkinson’s and Alzheimer’s diseases, therapeutic agents exist but only offer symptomatic relief [47]. Nevertheless, symptomatic relief does not meaningfully alter the disease progression.

Additionally, effective animal models that accurately reflect the condition of most neurodegenerative disorders in humans, including those with genetic origins, are currently unavailable [48,49]. Take HD as an example, various animal models, including rodents and non-human primates, have been established that replicate HD pathology and have been utilized extensively in gene therapy investigations [50]. Typically, these models are classified into nongenetic and genetic categories [51]. Chemically induced, nongenetic HD models involve neuronal damage through the use of excitotoxic substances, like quinolinic acid and kainic acid [52], or mitochondrial dysfunction agents, such as 3-nitropropionic acid and malonic acid [53]. However, these models fail to replicate the genuine pathological characteristics of HD. They do not produce mutant Huntingtin protein (mHTT). Additionally, HD is a hereditary condition causing a gradual progression of cell death, whereas in chemically induced models, cell death occurs rapidly. Consequently, their application is restricted to research focusing on neurorestorative and neuroprotective strategies. Moreover, several neurodegenerative disorders progress over extended periods, such as years or even decades, which poses significant challenges in replicating these timelines in animal studies.

Considering the broad heterogeneity in the phenotypes for each disorder, the proper classification of clinical trial participants into relatively homogeneous populations is difficult. Also, a rational strategy could involve defining endpoints that specifically address the non-psychiatric symptoms impacting quality of life; yet, they may be more readily quantifiable than more complex cognitive, social, or behavioral assessments. Nonetheless, a fundamental challenge in conducting clinical trials for treating neurodegenerative disorders is the absence of objective [54], reproducible treatment endpoints [55].

In summary, the major challenges in neurodegenerative diseases include the difficulty in distinguishing between different degenerative processes, the lack of effective treatment options, the absence of suitable animal models, the difficulty in properly classifying clinical trial participants into relatively homogeneous populations, and the inability to define clear endpoints for clinical trials, etc.

### 3.2. Opportunities for Drug Delivery

The diversity in neurodegenerative diseases introduces significant hurdles for pinpointing efficacious therapeutic targets and distinguishing individuals who are most likely to profit from targeted treatments. The scheduling, dosing, and administration of drugs are complex [56,57]. Moreover, while gene therapies exhibit considerable promise for neurodegenerative disorders, the low transfection efficiency, due to inherent issues like instability and negative charge, constrains their application [58]. Some cationic polymers can be used to overcome the challenges faced by gene drugs [59]. Prolonged exposure to drugs with side effects can be mitigated by using drug delivery systems in the nano and micron size range, which extend the release of active substances, overcome the BBB, improve patient compliance, and reduce side effects.

Currently, there are many strategies to fabricate drug delivery systems, including passive targeting strategies that rely on the enhanced permeability and retention (EPR) effect to accumulate drugs in diseased tissues, and active targeting strategies which use ligands, antibodies, or other molecules to specifically seek out and bind to target cells or tissues. The most frequently used drug delivery systems for passive targeting in the brain are liposomes [60]. Active targeting strategies mainly depend on antibody-mediated and receptor-specific delivery, wherein ligands are linked to nanoparticle surfaces to engage with particular receptors [61].

Additionally, responsive release mechanisms are employed to control the timing and rate of drug release based on environmental cues such as pH, temperature, or enzymes [62]. Multimodal imaging and therapeutic approaches are integrated into these systems to allow for both diagnostic imaging and treatment, providing a comprehensive solution for improved patient care and outcomes [63].

Enhanced intake of antioxidants has been shown to delay the onset of neurodegenerative disorders and improve the quality of life for those diagnosed with these diseases [64]. The current delivery systems for antioxidants mainly employ liposomes, polymeric nanoparticles, inorganic particles, hydrogels, and other types of delivery systems [65]. CD-based delivery systems belong to the category of polymeric nanoparticles. Antioxidants can be encapsulated inside or adsorbed onto the surface of CD-based delivery systems. By incorporating target molecules into delivery systems, antioxidants can exert their effects at specific sites. An ideal delivery system should feature high loading efficiency, accurate targeting, biodegradability, and biocompatibility [66,67]. As an example, resveratrol exhibits strong lipophilic, antioxidant properties. The impact of its complexation with CDs on antioxidant activity has been explored in numerous studies. Lucas-Abellán et al. examined the effect of resveratrol complexed with HP-β-CD on antioxidant capacity [68]. The antioxidant efficacy of resveratrol was influenced by its complexation, as CDs function as a controlled-release reservoir, shielding resveratrol from rapid oxidation by free radicals. Consequently, the antioxidant activity is extended and achieves its peak when all the resveratrol is complexed. However, findings that contradict those reported by Lucas-Abellán et al. were presented in another study regarding the impact on antioxidant activity [69]. In this work, the authors assessed the scavenging capacity of both free and complexed resveratrol at identical concentrations. According to their results, the differences in scavenging capacity between free and complexed resveratrol were minimal, indicating that the inclusion process had a negligible effect on antioxidant activity.

To sum up, the versatility of CDs allows for modifications that enable site-specific drug release, thereby reducing systemic side effects and improving therapeutic outcomes. The development of multifunctional CD complexes that combine diagnostic and therapeutic capabilities (theranostics) represents a promising area for future exploration, especially in the challenging realm of neurodegenerative disorders.

## 4. Construction of Cyclodextrin-Containing Drug Delivery Systems for Neurodegenerative Disorders

### 4.1. Alzheimer’s Disease (AD)

AD is a progressive brain disorder that worsens over time [70]. Approximately 1% of individuals aged between 50 and 70 years old experience AD, and the prevalence rises to 50% for those over 70 years old [71]. It is marked by the accumulation of specific proteins, including Aβ. As AD progresses, a specific decline in cholinergic neurons is observed, suggesting involvement of the acetylcholine neurotransmitter in memory and cognitive brain regions [72]. AD leads to brain atrophy and the eventual death of brain cells. It is the most frequent cause of dementia, which involves a gradual decline in memory, cognition, behavior, and social abilities.

Donepezil, rifampicin, galantamine, and acetylcholinesterase inhibitors are frequently used to increase the bioavailability of acetylcholine by preventing its breakdown [73]. Other proposed treatment options include the use of anti-inflammatory drugs, antioxidants, and non-steroidal anti-inflammatory medications [74], and monoclonal antibodies for immunotherapy, such as bapineuzumab and solanezumab [75].

**Figure 2:**
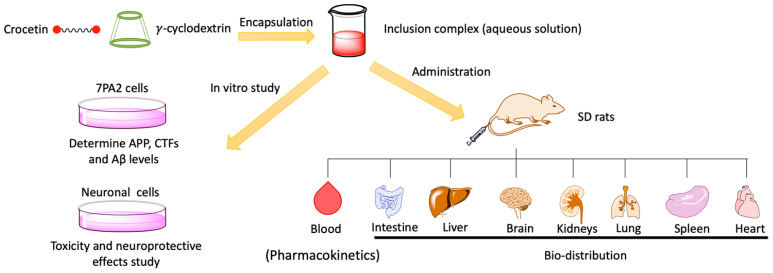
Schematic illustration of CRT-γ-CD for AD research. Reproduced with permission from [77].

Crocetin (CRT), an active compound derived from the fruits of gardenia (*Gardenia jasminoides* Ellis) and the stigmas of saffron (*Crocus sativus* L.), can decrease the generation of various neurotoxic molecules, inhibit Aβ fibril formation, destabilize pre-existing Aβ fibrils, and enhance Aβ degradation [76]. Wong et al. utilized γ-cyclodextrin (γ-CD) as a drug delivery system to enhance the solubility and bioavailability of CRT (Figure 2) [77]. The encapsulation efficiency of the system exceeded 95%, suggesting it is cost-effective and suitable for large-scale industrial production. In addition, the synthetic route did not require the use of organic solvents, thus avoiding potential toxicity, and in the toxicity assessment, it was confirmed that CRT, γ-CD, and the CRT-γ-CD were safe for both neuronal cells and AD model cells.

Park et al. investigated the impact of reactive oxygen-sensitive nanoparticles loaded with memantine for AD [78]. The nanoparticles were synthesized via the conjugation of succinyl β-cyclodextrin (bCDsu) with thioketal diamine and memantine (MEM) with thioketal carboxylic acid. These two components were then combined to produce bCDsu-MEM conjugates. In biodistribution studies, the brain uptake exhibited the highest fluorescence intensity. In oxidative stress studies, SH-SY5Y and U87MG cells expressed a time-dependent increase of the NMDAR1 receptor when exposed to reactive oxygen species, but this was counteracted by introducing memantine conjugates as a treatment.

**Figure 3:**
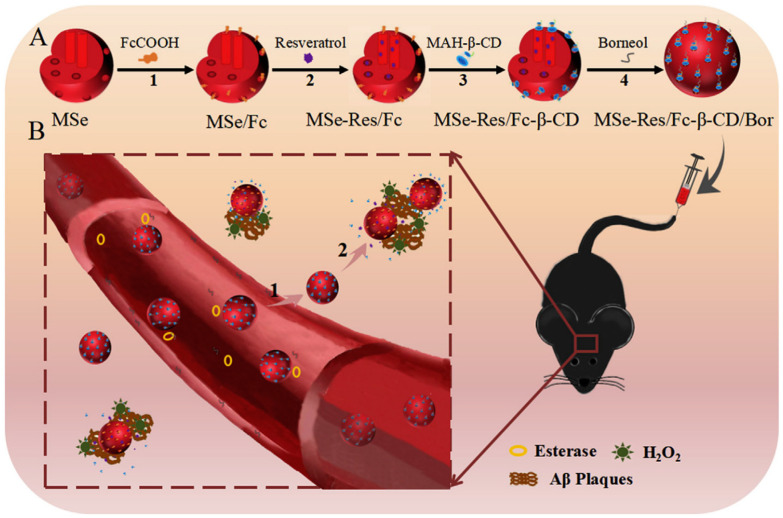
Schematic illustration of MSe-Res/Fc-β-CD/Bor for AD research. (**A**) The synthesis of MSe-Res/Fc-β-CD/Bor. 1. MSe modified with ferrocene. 2. Resveratrol encapsulated within MSe/Fc. 3. β-CD modified with MAH enclosing MSe-Res/Fc. 4. Borneol attached to the nanoparticle surface via ester linkage. (**B**) MSe-Res/Fc-β-CD/Bor in vivo. 1. MSe-Res/Fc-β-CD/Bor released borneol and crossed the BBB. (2) MSe-Res/Fc-β-CD/Bor selectively targeted amyloid plaques, achieving resveratrol release triggered by H_2_O_2_. Reproduced with permission from [79].

Sun et al. reported a novel mesoporous nano-selenium (MSe) release delivery system (MSe-Res/Fc-β-CD/Bor) based on borneol (Bor)-targeting, β-cyclodextrin nanovalves (Fc-β-CD) loaded with resveratrol (Res) (Figure 3) [79]. Previous experiments showed that MSe-Res/Fc-β-CD/Bor initially releases Bor through interaction with blood or intracellular esterases, enabling the nanosystem to cross the BBB. Following this, Fc-β-CD opens in response to redox (H_2_O_2_) at the lesion site, releasing Res. It was demonstrated that MSe-Res/Fc-β-CD/Bor inhibits the aggregation of Aβ proteins, alleviates oxidative stress, and suppresses tau hyperphosphorylation, while protecting nerve cells and improving memory impairment in APP/PS1 mice. Interestingly, compared to rivastigmine (Riv) alone, MSe/Fc-β-CD/Bor loaded with Riv showed improved pharmacokinetic properties. These findings indicate that MSe-Res/Fc-β-CD/Bor could be a promising therapy for AD.

The above studies provide some useful information for pharmaceutical research for AD therapy, and offer insights for the overall advancement of CD-based drug delivery systems by altering the systems to enhance drug transport across the BBB more effectively for AD therapy.

### 4.2. Parkinson’s Disease (PD)

Second to AD in prevalence among the neurodegenerative diseases, PD is etiologically linked to the malfunctioning folding and accumulation of α-syn proteins, causing neuronal death prominently in the dopaminergic nerve cells located within the substantia nigra [80].

Currently, only symptomatic treatments are available for PD, aimed at alleviating or preventing associated symptoms and enhancing dopamine action duration. Dopaminergic agonists, such as levodopa and carbidopa, are commonly used [81]. Levodopa functions as a dopaminergic agonist while carbidopa inhibits peripheral metabolism.

Barros et al. aimed to enhance the applicability of levodopa (L-DOPA) using CDs in a controlled drug delivery system for PD [82]. Various formulations in aqueous solution were proposed and characterized to understand molecular interactions. Two CDs were chosen, β-CD and its derivative, HP-β-CD, to evaluate the impact of substituent groups on L-DOPA solubility and interactions. The behavior of L-DOPA in the presence of these CDs was assessed through mutual diffusion coefficients, volumetric, and viscosimetric properties, providing insights into the potential of these systems for controlled drug delivery.

**Figure 4:**
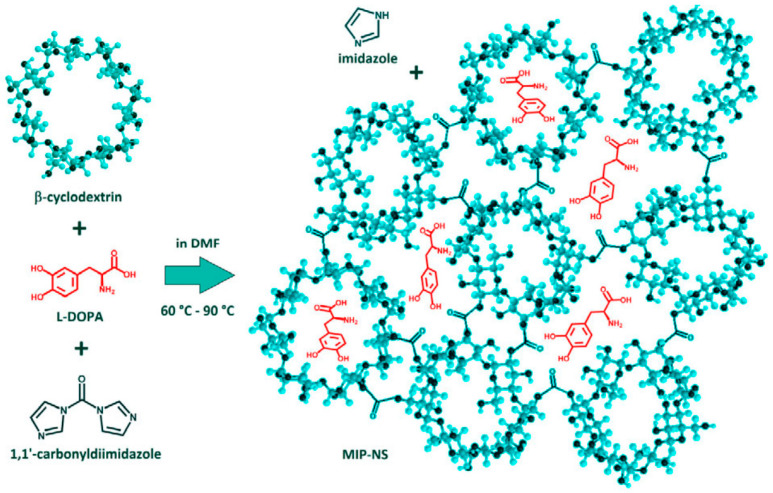
Schematic illustration of the MIP-NS synthesis process. Reproduced with permission from [83].

Trotta et al. focused on synthesizing a new type of CD-based nanosponge, using molecular imprinting technology to create molecularly imprinted nanosponges (MIP-NSs) for the controlled and prolonged release of L-DOPA (Figure 4) [83]. L-DOPA is unstable and prone to degradation when exposed to light or in aqueous solutions, which can diminish its therapeutic benefits. The synthesis process involved cross-linking β-CD with 1,1′-carbonyldiimidazole in N, N-Dimethylformamide, with L-DOPA as the template molecule. Characterization techniques included thermal gravimetric analysis, differential scanning calorimetry, and Fourier transform infrared spectroscopy to analyze the interactions between L-DOPA and the nanosponge structures. Quantitative nuclear magnetic resonance spectroscopy determined the amount and affinity of L-DOPA entrapped within the nanosponges. High-performance liquid chromatography was used to assess the in vitro release kinetics of L-DOPA from the nanosponges. The results showed that MIP-NSs provided a slower and more prolonged release profile compared to non-imprinted nanosponges. Importantly, no degradation of L-DOPA was observed within the MIP-NSs during long-term storage at room temperature. This indicates that β-CD-based MIP-NSs are a promising drug delivery system for protecting L-DOPA and achieving its sustained release, which could be particularly beneficial for oral administration.

Saitani et al. developed advanced drug delivery systems incorporating poloxamer 407, a non-ionic surfactant (Tween 80), and CDs (M-β-CD or HP-β-CD) for the potential nasal administration of ropinirole to treat PD [84]. The hybrid systems were fabricated using the thin-film hydration technique. They found that the drug release was diffusion-controlled and showed a gradual increase throughout the experiment. An ex vivo permeation study through rabbit nasal mucosa demonstrated superior performance of the hybrid systems compared to a ropinirole solution. The promising results in terms of drug release and mucosal permeation suggest that these hybrid systems could serve as effective platforms for targeted nose-to-brain delivery of ropinirole, potentially benefiting the treatment of PD.

Truzzi et al. chose β-CD and its hydrophilic variant, HP-β-CD, as potential carriers for geraniol (GER) to facilitate its nose-to-brain administration [85]. The biocompatibility of geraniol inclusion complexes with nasal tissues, along with drug bioavailability in cerebrospinal fluid (CSF), was evaluated in rodent models. Studies confirmed that stable 1:1 GER-CD complexes could be formed. The GER-HP-β-CD-5 and GER-β-CD-2 complexes showed similar characteristics. Although both complexes enabled safe and direct GER delivery to the CNS, the GER-β-CD-2 complex demonstrated a greater capacity for releasing GER into the CSF.

In summary, these results highlight the significant role of CD as a promising therapeutic agent for enhancing α-syn aggregate degradation during disease progression.

### 4.3. Multiple Sclerosis (MS)

MS is an autoimmune disease that affects the CNS, including the brain and spinal cord. MS has a genetic predisposition, triggered by a combination of unidentified environmental factors, likely including viral infections [86]. Globally, approximately 2.8 million individuals are affected by MS, including young people, and it is a primary cause of disability in this age group [87]. Typical symptoms may include numbness, muscle weakness, vision problems, balance issues, dizziness, bladder control difficulties, tiredness, and depressive moods [88].

Currently, there is no cure for MS. Despite the reporting of numerous structurally diverse MS drug candidates in the literature, two primary classes of compounds, based on their specificity, are utilized for MS treatment: immunomodulators and immunosuppressants [89]. Filippini et al. reviewed 44 trials, involving 17,401 participants who were randomized [90]. They found that natalizumab and interferon beta-1a (Rebif) demonstrated superior outcomes within a 24-month period compared to placebo. However, these therapies are linked with serious adverse events over the long term, potentially leading to an unfavorable benefit–risk profile. Interferon beta-1b (Betaseron) and mitoxantrone likely reduce the likelihood of relapses, supported by moderate-quality evidence when contrasted with placebo. The efficacy and safety balance for azathioprine remains unclear. Nonetheless, it may effectively decrease relapse rates and disability progression over 24 to 36 months relative to placebo. Additionally, various cell-based therapies have now been introduced for MS treatment [91]. Phase I trials using tolerogenic dendritic cells (TolDCs), mesenchymal stem cells (MSCs), and Tregs in MS patients demonstrated safety and good tolerance, without significant adverse reactions. MSCs exhibit strong immunomodulatory impacts, TolDCs pulsed with self-antigens induce tolerance, whereas Tregs, including newer CAR-Tregs, target the disease’s root cause—the autoimmune reaction to CNS myelin—while preserving protective immunity. Furthermore, though less investigated, certain cell-based treatments display neuroprotective and reparative capabilities. Such cells have yielded encouraging outcomes in experimental setups, with some like hfNSCs, MSC-NPs, and hESCs currently under clinical evaluation.

Strategies enhancing access and accumulation within the CNS might provide MS treatments with new chances to address the neuro-inflammatory and neurodegenerative processes characteristic of progressive forms of MS [92]. Alternatively, an intriguing non-invasive method for delivering drugs directly to the CNS, bypassing the BBB, involves utilizing the nose-to-brain (N2B) pathway [93]. Permeation enhancers, such as CDs, surfactants, and saponins, can increase membrane fluidity, tight junction permeability, and/or generate hydrophilic pores, which are strategies to enhance N2B transport and further support drug delivery to the CNS [94]. To assess how adding a mucoadhesive component influences the absorption of drugs into the brain, Khan et al. compared brain levels of the drug post intravenous administration, intranasal application as a solution lacking chitosan or CDs, and intranasal delivery as a solution containing 1% chitosan and 5% HP-β-CD [95]. They found that CDs might have additionally enhanced the brain concentration by boosting the drug’s permeability through the tight junctions of the nasal epithelium.

### 4.4. Amyotrophic Lateral Sclerosis (ALS)

ALS is a devastating condition that results from motor neuron degeneration. Similar to other major neurodegenerative disorders, developing therapies poses significant challenges for various reasons [96]. In approximately 97% of ALS patients, ALS involves TAR DNA-binding protein 43 proteinopathy [97]. However, there is also significant pathological heterogeneity: ALS caused by mutations in the SOD1 (Cu-Zn superoxide dismutase) and FUS (fused in sarcoma) genes does not involve TDP-43 proteinopathy, although cytoplasmic protein aggregates of varying composition are present [98]. Additionally, the most common genetic subtype of ALS, linked to intronic hexanucleotide GGGGCC expansions in the chromosome 9 open reading frame 72 gene, exhibits TAR DNA-binding protein 43 mislocalization but also contains additional p62-positive protein aggregates due to pathological dipeptide repeat proteins [99].

Linda et al. indicated that HP-β-CD fails to provide any therapeutic advantage in SOD1G93A mice. Nonetheless, the lack of harmful effects is insightful, considering the frequent application of CDs as complexing agents in various pharmaceuticals, their potential for independent therapeutic use, and the newly recognized link between dyslipidemia and ALS advancement [100].

### 4.5. Huntington’s Disease (HD)

HD is a rare autosomal dominant neurodegenerative disorder triggered by the expression of a toxic Huntingtin (HTT) protein. HD affects cognitive and motor abilities, with a prevalence of five to seven cases per 100,000 people [101]. Furthermore, evidence indicates that alterations in cholesterol metabolism may contribute to disease progression, while increased caveolin-1 expression plays a crucial role in cholesterol transport and balance [102,103].

Godinho et al. explored the use of short interfering RNAs (siRNAs) to silence the mutant HTT protein [104]. The main challenge in siRNA-based approaches is the lack of effective and non-toxic delivery systems for siRNA transport to the CNS. They evaluated modified amphiphilic β-CDs as innovative siRNA carriers. They demonstrated that CDs formed stable nanoscale particles in artificial CSF and were capable of reducing HTT gene expression in rat striatal cells (ST14A-HTT120Q) and human HD primary fibroblasts. Minimal toxicity was observed with CD.siRNA nanoparticles. Sustained knockdown effects were evident in the striatum of the R6/2 mouse model of HD after single direct injections of CD.siRNA nanoparticles. Repeated brain injections of CD.siRNA complexes led to selective improvements in motor deficits in the mouse model.

Mendonça et al. developed three CDs with unique chemical compositions. Their efficiencies were evaluated as potential carriers for antisense oligonucleotides targeting HTT. Outcomes from experiments using striatal neurons and HD-patient-derived fibroblasts showed that modified γ-CDs demonstrated superior uptake efficiency and effectively reduced mHTT at both the protein and allele levels. Integrating the brain-targeting peptide rabies virus glycoprotein (RVG) into the modified γ-CDs resulted in enhanced reduction of the mHTT protein and HD-causing effects [105].

The application of histone deacetylase (HDAC) inhibitors has been observed to counteract lethality and photoreceptor degeneration in Drosophila models of polyglutamine diseases. To delve deeper into the therapeutic potential of HDAC inhibitors, researchers conducted preclinical experiments using suberoylanilide hydroxamic acid (SAHA), a powerful HDAC inhibitor, in the context of the R6/2 HD mouse model. Their findings revealed that SAHA is capable of crossing the BBB and increasing histone acetylation levels in the brain. It was also noted that SAHA could be effectively administered orally through drinking water when it was complexed with CDs. The study showed that SAHA led to a significant improvement in motor impairments experienced by R6/2 mice, thereby reinforcing the viability of pursuing HDAC inhibitors as a therapeutic approach for HD [106].

**Figure 5:**
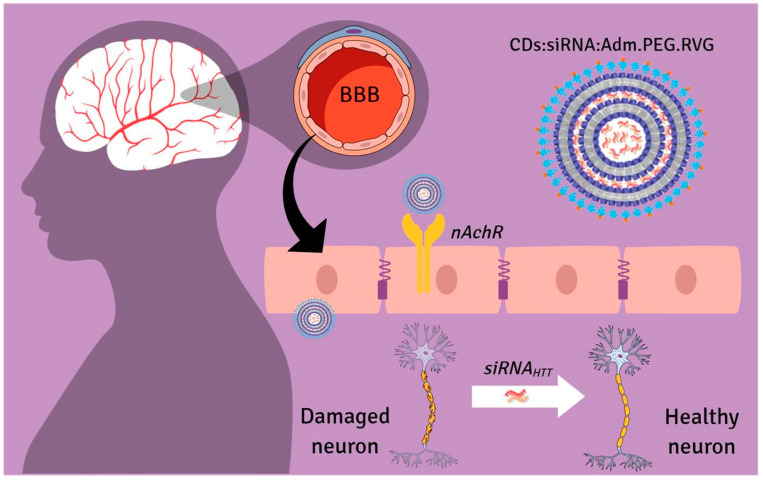
Schematic illustration of CDs loaded with siRNAs, and conjugated with RVG peptide, which could cross the BBB, target HTT, and restore damaged neurons to a healthy state [107].

Mendonça et al. developed an innovative delivery system that utilized modified CDs, loaded with siRNAs targeting HTT, and conjugated with the brain-barrier-crossing peptide RVG (Figure 5) [107]. The results from an in vitro BBB model showed that this formulation can effectively cross brain endothelial cells, release the encapsulated siRNAs into the neuronal cell cytoplasm, and mediate the downregulation of HTT. Overall, this CD platform showed broad application prospects as a means of delivering siRNA-based treatments for HD and potentially for other conditions with genetically validated targets in the CNS.

## 5. Conclusions

The investigation of CD-containing drug delivery systems spans several decades, yielding substantial insights into their efficacy in enhancing the solubility and bioavailability of pharmaceutical compounds [108]. Despite extensive research in diverse therapeutic areas, the application of CDs specifically for the treatment of neurodegenerative disorders remains a largely underexplored domain. The intricate nature of the BBB presents a significant obstacle to drug delivery [109,110]; however, CDs demonstrate potential as a vehicle capable of navigating this complex physiological barrier.

Preliminary studies suggest that CD-based formulations can facilitate the transport of therapeutic agents to the brain, yet the field lacks comprehensive clinical validation and standardized methodologies tailored for neurological conditions. The development of more effective drug delivery strategies is essential for addressing the complexities of neurodegeneration. CD-containing systems, through their ability to encapsulate and protect drugs, may serve as a pivotal tool in advancing treatments for conditions such as AD and PD.

Future research endeavors should focus on refining CD formulations to ensure effective penetration of the BBB, enhance the stability of neuroactive drugs, and achieve targeted delivery to specific brain regions. Comprehensive preclinical evaluations and well-designed clinical trials are crucial for establishing the safety and efficacy of these novel delivery systems. Through such efforts, the groundwork can be laid for transformative therapies aimed at addressing the urgent needs of patients with neurodegenerative disorders.

In summary, while CDs have been extensively studied in the context of drug delivery, their specialized application in neurology represents a fertile area for further exploration. Harnessing the unique properties of CDs holds promise for the development of advanced therapeutic approaches that may redefine the treatment landscape for neurodegenerative diseases.

## Data Availability

Not applicable.
